# Development and content validation of a sunlight exposure diary in patients with erythropoietic protoporphyria

**DOI:** 10.1186/s41687-023-00655-y

**Published:** 2023-11-20

**Authors:** Susan D. Mathias, Laurie Burke, Hilary H. Colwell, George Mensing, Will Savage, Hetanshi Naik

**Affiliations:** 1 Health Outcomes Solutions, Palm Beach Gardens, FL USA; 2 LORA Group, Normal, IL USA; 3 Disc Medicine, Watertown, MA USA; 4 Department of Genetics, Stanford University School of Medicine, Palo Alto, CA USA

**Keywords:** Erythropoietic protoporphyria, Patient reported outcomes, Quality of life, Sun exposure diary

## Abstract

**Background:**

Erythropoietic protoporphyria is a rare, inherited disorder presenting in early childhood with severe, painful phototoxicity. EPP has significant impacts on health-related quality of life, though there is variable disease severity. Accurately capturing how much time individuals with EPP can spend outdoors before they develop symptoms is critical to understanding HRQoL and measuring therapeutic response. Therefore, the goal of this study was to develop a comprehensive and content valid sun exposure diary to assess the efficacy of new therapies in individuals with EPP.

**Methods:**

Qualitative interviews were conducted with adult and adolescent EPP participants, as well as five clinical experts, to obtain their input on the content of an existing sun exposure diary. Revisions to the diary were made based on evidence generated in cognitive debriefing interviews analyzed in eight consecutive groups of EPP participant.

**Results:**

Interviews were conducted with 17 adults and 6 adolescents with EPP. The average age of adults was 40 years and of adolescents was 14 years. Clinical experts thought the original diary needed clarification on the description of symptoms, how time outdoors was captured, and the distinction between direct vs. indirect sunlight. Participants with EPP also noted these items needed revision, and that the distinction between prodromal symptoms and full reaction symptoms should be clarified. In the final diary version, participants with EPP found most items to be clear and easy to complete/think about. Seventy-six percent of participants (13/17) asked thought the diary was easy to complete. The remainder thought the majority of the diary was easy to complete with the exception of select questions.

**Conclusions:**

Evaluating a new treatment for EPP requires accurately capturing time in sunlight and symptoms in this unique disorder. The newly developed sun exposure diary is content valid and can be used to assess important aspects of symptoms and daily life and therefore evaluate clinically meaningful therapeutic response.

## Background

Erythropoietic protoporphyria (EPP) and X‐linked protoporphyria (XLP), collectively referred to as EPP, are rare, inherited photodermatoses that generally present in childhood with severe, painful phototoxicity [1, 2]. Symptoms after sun exposure can include a prodromal syndrome with tingling, burning, and/or itching that may progress rapidly to severe pain, erythema, and swelling. Severity is variable, but most patients experience symptoms within 30 min of sun exposure, and severe phototoxic reactions can last for several days [1–3]. There is only one approved treatment for adults with EPP in the US and the European Union, Scenesse (afamelanotide), a subcutaneously administered α-melanocyte stimulating hormone analogue, but no therapies are currently available for pediatric patients [4].

Daily life is significantly impacted for individuals with EPP [4–10] not only because of the symptoms of phototoxicity, but also because the disease necessitates avoiding sunlight as much as possible. Accurately tracking sunlight exposure is important for evaluating new therapies and pain-free light exposure time is the precedented, clinically meaningful endpoint used to support afamelanotide’s marketing authorization in the US and European Union. However, the sun exposure diary utilized in those trials had some limitations: iterative patient feedback was not solicited during its development, the Likert pain scale superimposed qualitative categories of mild/moderate/severe, and the diary was deployed on paper.

The goal of this study was to conduct in-depth, qualitative interviews with adults and adolescents with EPP in order to evaluate and modify the content of a sunlight exposure diary where necessary to document evidence of content validity.

## Methods

Qualitative interviews were conducted from March to June 2022, and the study was approved by the WCG Institutional Review Board.

### Recruitment

Potential participants were referred from the United Porphyrias Association (https://www.porphyria.org). Adult participants provided written consent; parents consented for pediatric participants and pediatric participants assented.

### Participants

Full inclusion criteria are described elsewhere [11]. Briefly, participants with EPP were ≥ 12 years old, had a confirmed diagnosis of EPP, and resided in the US or Canada. If participants were treated with afamelanotide, treatment was initiated no more than three months prior to enrollment. Participants completed a demographic survey and received a $150 Amazon gift card after participation was complete.

### Interviews

Initial interviews were conducted with 5 individuals with EPP and 5 clinical experts that treat EPP, using the same diary that was used in the afamelanotide clinical studies [4]. In those studies, phototoxic pain for each day was recorded by answering the question, “Have you experienced any reactions to light today?” (Yes/No) and indicating the pain level on a scale from 0 to 10 (‘no pain’ to ‘worst imaginable’). Subjects also recorded the amount of time spent outdoors in either direct sunlight or shade between 10:00 to 18:00 hours [4]. Results from those interviews were used for initial modifications to the diary.

One-on-one interviews were conducted using online video conferencing by an experienced health service researcher. Transcripts were coded and analyzed using a qualitative software, MAXQDA. The diary content was revised iteratively based on eight subsequent waves of interviews (for a total of 23 interviews [*n* = 17 with adults and *n* = 6 with adolescents]). The first 5 interviews with individuals with EPP included open-ended concept elicitation (CE) questions only. CE questions are used to better understand the symptoms and impacts experienced by individuals with EPP. Examples of CE questions for sun exposure included “How much time do you typically spend outside each day in direct sun?” and “When you do go outside, what type of clothing do you typically wear?”. All subsequent interviews included both CE and cognitive debriefing (CD) of the modified sunlight exposure diary. CD questions are used to obtain specific feedback about the content, clarity, and relevance of the draft sunlight exposure diary. Results of the CE portion are described elsewhere [11]. The results of the CD portion of the interviews focusing on the sun exposure diary are reported here.

## Results

### EPP participant characteristics

Seventeen adults and six adolescents with EPP participated. There were approximately equal numbers of males (52%) and females (48%), the majority were White (94%) and non-Hispanic (88%). Most adults had a college degree or higher (71%), worked full-time (71%), were married or living with a partner (71%), and the mean age was 40 years. Most adults were not receiving any treatment, 47% previously received afamelanotide, and 13% were currently receiving afamelanotide. On average, the adolescents were 14 years of age and had a high school education or less. Additional participant characteristics are described in Mathias et al [11].

### Cognitive debriefing of sunlight exposure diary

#### Clinical expert interviews

Feedback from clinical experts on the original diary [4] included:

- 4/5 thought adding instructions to the diary would be helpful
- Three experts thought individuals with EPP would understand the phrase “reactions to light,” but two did not think the wording in the pain item was clear (whether referencing pain associated with a reaction or pain associated with prodromal symptoms).
- Two felt it would be helpful to know which specific prodromal symptoms were experienced.
- Two indicated it might be confusing thinking about time outside if this includes exposure to sunlight while in a car or sitting by a window.
- One thought increments of 15 min were too long to consider and suggested using start/ stop times instead. Another liked the 15-min intervals and felt it would be too difficult to use start/stop times. One expert would expand the range from 7:00 am until 9:00 pm because of the potential for sunlight exposure during those hours, especially during certain months of the year.
- Two did not think the question about time in shade was relevant.
- Most experts agreed it was more appropriate to ask about “today” versus “the past 24 h.”

### EPP participant interviews

Each participant was interviewed only once, and therefore only reviewed one version of the diary. Most participants were able to accurately paraphrase the items in Version 1 of the Diary (range: 80–100%). However, the items of “any reactions to light today” and “time in direct sun and time in shade” were unclear. Some participants (40%) were confused by the use of military time and some (40%) did not know how to answer if sun exposure was less than 15 min.

Based on results from these initial interviews with clinical experts and participants with EPP, the original diary was revised (Version 2). In general, participants were able to accurately paraphrase all items in the diary (range: 80–100%). Most found the items to be clear (range: 60–100%) and could rephrase the question accurately in their own words. Of those who found it unclear, fifty percent found the question “did you have early warning symptoms or a painful reaction” to be confusing because some individuals experience pain as an early warning symptom. Sixty-seven percent were unsure how to respond to the question about time in shade.

In Versions 3 and 4b, participants were able to correctly paraphrase the instructions and all items. Most questions were easy to think about, with one exception. For the question about total time in sunlight, one participant thought it was unnecessary to include one start/stop time for each hour since she would never leave her home more than one time each day.

In Versions 5–7 minor revisions were made between each version. Most participants were able to accurately paraphrase each item (range: 67–100%). Most (67–100%) thought the questions were easy to complete/think about. With one exception, participants found the questions to be clear. One participant would add “whether you experienced a reaction or not” to the question about “whether you were exposed to sunlight.”

Version 8 of the Diary was debriefed with two adolescents. Both were able to correctly paraphrase the instructions and all items. With some exceptions, participants found the items to be clear. One adolescent did not know what “severity” meant and was not able to describe the word “moderate” but could figure out the meaning within the context of the question and other response options. For the question about exposure to sunlight, one suggested adding “sun through a window or sunlight through a car window” as examples of indirect sunlight.

During the final round of interviews, all participants were able to correctly paraphrase the instructions and all questions. Participants found most items to be clear and easy to complete/think about. Table 1 summarizes the key changes from the original to the final diary, and Table 2 includes sample final questions. A copy of the diary is available upon request from the authors.

**Table 1:** Key revisions made to sunlight exposure diary based on cognitive debriefing interviews

Original wording of questions	Key revisions
No instructions provided	Added instructions
Have you experienced any reactions to light today?	Revised to ask about early warning symptoms and full reactions
N/A	Added question to ask whether the reaction was new or continuation from prior day
If yes, please indicated on the scale below how bad your pain was from the reaction	Revised to ask about pain from a full reaction at its worst
	Revised response options (removed some of the labels on the pain scale)
Did you spend any time outdoors today?	“Direct or indirect” added to clarify that respondent should consider all sources of sunlight
If yes, please enter the time period that you were in direct sunlight (Each box represents 15 min)	Question revised to ask about minutes in sunlight (direct and indirect) between 6:00 am and midnight
	Revised from recording in 15 min blocks to exact minutes
	Tested a version recording stop/start times but based on feedback total number of minutes used instead
If yes, please enter the time period that you were in shade	Item omitted—respondents did not find it relevant
N/A	Added questions about symptoms experienced (e.g., “feelings of warmth”, “sensitivity to touch”, “tingling”, “burning”, etc.) and the severity of each symptom (mild, moderate, severe)
Questions use 24-h recall period	Changed to insert day/date (e.g., Monday, February 27)

**Table 2:** Sample items from final sunlight exposure diary

Sample items	
*On [insert day of the week and date], did you have …. (check all that apply)*	
Early warning symptoms from sunlight	
A full reaction from sunlight	
I did NOT have early warning symptoms or a full reaction from sunlight	
*Were you exposed to sunlight (direct or indirect) on [insert day of the week and date]?*	
Yes	
No	
If YES, please indicate how much TOTAL time you were in sunlight (direct and indirect) during each of the following time periods on [insert day of the week and date]. For example, if you went out for 5 min at 1:15 pm and 10 min at 1:45, you would record 15 min for the afternoon hours of 1:00–2:00 pm. You will also be asked to indicate whether you experienced any early warning symptoms during that time	

### Meaningful change to level of pain

Participants were asked how much change in the level of pain would be meaningful if they were in a clinical study for an EPP treatment, on a 10-point scale ranging from 0 (no pain) to 10 (worst pain imaginable). Responses ranged from 2 to 7 points, with most saying ~ 5 points would be meaningful.

### Recall period

Participants who completed Versions 1–4 were asked how easy or difficult it was to think about the past 24 h (from midnight to 11:59 pm on a specific day) when answering the questions. Eighty percent (*n* = 4 of 5) thought it was an easy timeframe to consider. One participant said it could be difficult. The diary was then revised to include a specific day of the week and the date (versions 5–8). All participants (*n* = 5) thought it was easy to recall back for a specific date.

### Ease/difficulty of completing diary

Seventy-six percent of participants (*n* = 13 of 17) thought it was easy to complete the diary. Two participants thought it was easy except for the question about time in sunlight by hour. One participant said between easy and medium, and one said easy except for a few words (adolescent who had difficulty with “severity” and “moderate”). Ninety-four percent of participants (*n* = 15 of 16) thought the formatting was clear.

## Discussion

The goal of this qualitative research was to confirm the content validity, relevance, and clarity of a sunlight exposure diary in individuals with EPP. The diary was cognitively debriefed during a total of 23 combined CE/CD interviews with adults and adolescents.

Individuals with EPP and clinical experts, who are also researchers, were asked to review and complete (*n* = 5 individuals with EPP only) the original version of the sunlight exposure diary. Based on their feedback, it was determined that revisions were needed. Revisions included providing more detailed instructions, allowing respondents to record time in sunlight in exact minutes (versus 15-min increments), further differentiating early warning symptoms (prodrome) from full reactions, assessing common symptoms experienced by individuals, omitting reference to time in shade, and including reactions from indirect sunlight to capture scenarios like sun coming through a window of a car or home, reflected off of surfaces.

During nine total waves of interviews, and following several additional modifications (see Table 1), cognitive debriefing revealed that individuals with EPP were able to understand and complete the sunlight exposure diary with ease. Problematic questions/phrasing were identified, and minor revisions were made following each wave of interviews. In general, the final sunlight exposure diary was found to be clear, comprehensive, and relevant.

Data from this diary will result in a detailed documentation of sun exposure and associated symptoms, which will be used to analyze total pain-free time in light as well as frequency and severity of symptoms. Though subject to recall bias, encouraging daily completion can minimize this. In addition, data collected from this diary is one element of trial participation and the patient experience, and can be complemented with objective measures such as light dosimetry. There is also inherent individual variability in sun exposure due to geography, seasonality, and clothing that can affect the diary responses. This can be accounted for, in part, by capturing zip codes for participants, and these variables are less confounding if treatment effects on sunlight exposure are evaluated in a randomized controlled trial.

This study included a fairly heterogenous sample in terms of demographic characteristics and was geographically diverse. Limitations included a relatively small sample of adolescents and a lack of racial/ethnic diversity of participants.

This sunlight exposure diary was developed in accordance with FDA guidance and the first to be rigorously debriefed to ensure its content is relevant and readily understood with individuals with EPP. The new version of the diary can be considered content valid and will be valuable for use in clinical trials of new EPP treatments.

## Data Availability

The datasets generated and/or analyzed during the current study are not publicly available due to privacy concerns but are available from the corresponding author on reasonable request.

## Abbreviations

EPP: Erythropoietic protoporphyria
XLP: X-linked protoporphyria
HRQoL: Health related quality of life
TTP: Time to prodrome
CE: Concept elicitation
CD: Cognitive debriefing
